# The Cost of Hospitals with Medical Schools: King Edward's Hospital Fund for London Statistics—Third Notice

**Published:** 1915-01-23

**Authors:** 


### THE COST OF HOSPITALS WITH MEDICAL SCHOOLS.*

### King Edward's Hospital Fund for London Statistics.—Third Notice.

The one outstanding fact to be gathered from the summary of the position of the larger general hospitals set forth in the last article is that the hospitals of London with medical schools cost more to manage, both as regards in-patients and out-patients, than the other general hospitals, and this statement may be extended to the effect that they are also more expensive than any other group.

The average cost of seventeen larger general hospitals per occupied bed for 1913 was £84 11s. 2d.; no other group approaches this figure, and the only groups coming between £70 and £80 per bed are the smaller general hospitals and the hospitals for women. All the other medical institutions of London work out at below £70 per bed.

The bargain that our voluntary hospitals make with the medical profession—that in return for free services a certain status in the professional world is given—is an excellent one both for the hospitals and for their honorary medical staffs. The arrangement is so closely bound up with the voluntary system that any change would make radical alterations to that system, and would also intimately affect the organisation for teaching medical students and the position of specialists.

This bargain few would have arranged on a different basis. It must not, however, be thought that the possession of an eminent honorary staff is entirely costless to a hospital; it brings with it inevitable extra expenditure, for the more experienced the medical man, the more exacting are his requirements in respect of treatment. It is therefore a corollary to the proposition that as the hospitals with medical schools have larger staffs and the *personnel* belong to the cream of the profession, these hospitals may be expected to cost more to manage. The school itself, however carefully expenditure is separated, has some expenses, often indefinable, which do not occur in a hospital without a school. For instance, a large number of people use the building, and the general standing charges are consequently heavier. We must not, in considering these points, overlook the probability that increased financial support will accrue through the staff and school; especially is this the case when the hospital sends forth annually a stream of medical men who will always find a place in their affections for their training school.

When we examine our list of the seventeen great hospitals in order of cost of expenditure, we notice that certain outlying hospitals take pride of place in being the most economically managed. The reasons for this suggested in our review of the Report for 1912 were admittedly not entirely sufficient. We think that the position in the list of these institutions is due to the fact that they are not associated with medical schools, and not because there is some peculiar influence in relation to cost arising out of their geographical position. There are, of course, one or two hospitals without schools which come in cost above some of the teaching hospitals, notably the Metropolitan and the Great Northern Central, but even these rank in order of lowness of expenditure above seven or eight others.

On the whole the suggestion could be advanced for consideration that in future issues of the Report the hospitals with schools might with advantage be tabulated by themselves. Taking in this way the hospitals with schools for 1913, the average cost per bed was £88 9s. 2d. (instead of £84 11s. 2d., as quoted above), over £8 higher than the highest of any other group. The teaching institutions are thus given an advantage of about £4 per bed at the expense of the other, not necessarily smaller general hospitals, which, we suggest, should be considered apart. The cost per bed of the hospitals eliminated was £70 13s. 1d.; higher than some groups and lower than others, being, we should judge without elaborate calculation, about the average of the remaining groups put together.

Given accurate and consistent bookkeeping, the tabulated results are interesting and instructive. In carrying out the greater part of the instructions for preparing the accounts under the Uniform System, all that is required upon the part of the accountant is accuracy; but occasions arise when a choice as to under which heading certain expenditure should be allocated must be made on the responsibility of the head of the department issuing the accounts. We are not speaking for the moment of the separation of the in-patient and out-patient figures—that subject shall be dealt with later. Reference is now made to such points as the division of certain salaries between "Maintenance—Salaries and Wages" and "Administration—Management"; electric current charged for in the gross but to be divided by calculation between "Domestic" and "Surgery and Dispensary," and many other matters where discretion is needed.

### The Middlesex Hospital Cost Per Bed.

The lay officers of our voluntary hospitals are proud of their calling, and every inducement is given them to make themselves more and more efficient. They have frequent opportunities of comparing notes on subjects of mutual interest. In the main, therefore, the practice in respect of these discretionary matters should have a rough uniformity. But when we see in these tables an institution holding in two successive years such widely differing places as fifth and fifteenth in order of lowness of cost per occupied bed, one, failing to discover the reason by means of the disclosed factors, is tempted to inquire if hospital bookkeeping is really so consistent after all, or if we are at fault because the supplied data are insufficient. The instance we allude to is that of

* Articles I. and II. appeared on October 10 and December 12, 1914.

Middlesex Hospital, whose cost per occupied bed was in 1910 (King's Fund figures) £80 18s. 5d., jumped to £90 3s. 6d. in 1911, and fell to £75 13s. 4d. in 1912. The headings "Domestic" and "Salaries and Wages" accounted for most of the rise of 1911, during which year we notice that the cost of management (administration), apart from salaries and pensions, was doubled. When we come to the fall of 1912, the headings chiefly responsible show themselves again to be "Domestic" and "Salaries and Wages."

The allusion to this matter is in no way intended to involve any criticism of the management of the hospital; it is simply put forward to provide an illustration of where the collected statistics do not help us. Here, where we have a large rise in "Salaries and Wages" and a larger fall, some information as to the size of the staff (a fact which governs much of the expenditure under more than one or two headings) would be exceedingly useful in solving such a problem.

This information was asked for last year, and, strengthened by the above illustration of its utility, the request is renewed.

